# Pelvic Abscess

**Published:** 1867-02

**Authors:** Morton M. Eaton

**Affiliations:** Peoria, Ill.


ARTICLE XI.

### PELVIC ABSCESS.

BY MORTON M. EATON, M.D., of Peoria, Ill.

This article is written more as a caution to the use of pessaries in the treatment of prolapsus uteri, than with any hope of offering anything new on the treatment of "Pelvic Abscess," which, I believe, is well established. I may remark, however, that such cases are not very desirable, as the investigation and treatment of the case must be very embarrassing to both physician and patient, especially if the patient is a fine lady in the first walks of life, and you are called to take charge of the case after a good physician has been dismissed.

I will now relate a case which has been of interest to me from its rarity, I never having had but one case of the kind before.

Was called to see Mrs. I., of this city, wife of a merchant living on —— st., September 5th, 1866. Found that Dr.——, a very fine physician, so called, had been attending and treating her; first for "falling of the womb," in which he had used various hard pessaries, afterwards had treated her for ulceration of the os uteri (the prolapsus not being cured), then again applying the pessaries; the whole time of his treatment having extended over a year or more, nearly ever since the birth of her only child, some 15 months since, had been dismissed, and I was requested to take charge of the case, which I hesitated to do, because I had great respect for the physician that had been employed over a year and dismissed. But assented after urgent solicitation by the patient and her mother, who had been a patient of mine six years since, while suffering from some female complaint. I consented to examine the case.

I found her plethoric, aged 24 years, the mother of one child; now confined to her bed, where she had already been for over two weeks, complaining of severe pain in the pelvis, and in the hypogastric region. Pulse 110, skin dry, tongue with a white coat; defecation produced severe pain, and the erect posture could not be endured; micturition painful and desire frequent; abdomen tender. On attempting to make a digital examination per vaginum, I found all the parts so sensitive and so much inflamed and swollen I desisted, and contented myself with knowing that all the organs within the pelvis were highly inflamed, and that I had reason to fear the formation of a pelvic abscess. I ordered fomentations of hops over the external genetalia and hypogastric region, and gave opii. pulv. gr. ss., et hydrarg. chlo. mit. gr. j., every three hours.

*Sept. 7.* Discontinued the mercurial and continued the opiate, and on examination finding considerable swelling on one side of the anus, I changed the hops for a poultice of flax-seed meal, being now well satisfied an abscess had formed, and hoping to have it open externally in its most depending situation. But the swelling lessened the next two days under the application of the poultice, I thought possibly I would not have an abscess; but on making a digital examination per vaginum, which the subsiding of the inflammation permitted, I detected a large fluctuating tumor to the right side of the vagina and posteriorly to the same. I then used vaginal injections of warm water, hoping the abscess would open into the vagina, but I was disappointed; for on the next day, Sept. 12th, it opened into the rectum, and discharged profusely. I now gave the elix. of the valerianate of ammonia as an anodyne, and discontinued the opiate, and ordered an enema of warm water and soap twice a-day. Tried a compress in the vagina to press the sides of the abscess together and cause union, and prevent the accumulation of matter, but it could not be retained. The os uteri was very tender and very low in the pelvis. Applied an elastic abdominal supporter to the abdomen to press the bowels up, and take off the pressure of the bowels from the uterus, and used no treatment for about two weeks, except the supporter, the valerian to quiet the nerves, and gave potass iodid, 3 gr. doses, with syr. sarsaparilla three times a-day. At this time, Sept. 26th, she could sit up without suffering any bearing down pain, but on rising, a pouch was formed beside the rectum, and it being fluctuating, I was certain it contained matter, and feared fæcal matter besides. I accordingly opened the tumor with a sharp pointed bistoury, and considerable pus escaped, but nothing of a fæcal character. I now washed out the cavity with soft tepid water, and injected solu. iodine:—Iodine gr. v., potass iodid. gr. xij, aqua ℥j, once a-day, enjoining rest and a farinaceous diet for a week; when I directed her to sit up, and she found she could do so by using a soft pillow to sit upon. She still wore the abdominal supporter, and declared herself feeling better than she had for a year. I gave her elix. bark et ferri protox in ℥j doses, and used the iodine 10 gr. to the ℥j of water once in two or three days. The discharge constantly grew less and less, until Nov. 2d, when it ceased; Nov. 10th, patient discharged cured. By the constant use of the abdominal supporter, the uterus had regained its natural position without any direct treatment, (so called); although I claim that the taking off of the weight of the abdominal viscera from the uterus is the most philosophical treatment in some cases, and I believe this was one. Nov. 20th, saw Mrs. I. at my office, who requested an examination of the uterus to know that those *ulcerations* were cured. Agreeable to her request, I examined her with a bladed speculum, and found the os uteri perfectly healthy, and all the parts, so far as I could learn, were healthy also.

I told her she was "all right," needed no treatment, but to wear the abdominal supporter. Jan. 1st, I learned from Mrs. I. that she was still perfectly well, and had experienced no pain or trouble of any kind for a long time.

Now this patient had not been recently confined, or exposed to those causes which ordinarily produce this complaint; it is true that authorities state that these abscesses sometimes form without any known cause. But, whatever may have been the cause, in this case I am certain she was treated by authority for the prolapsus, etc. But I do feel disposed to discourage that too common practice with some of finding ulceration and using caustics; and prolapsus and using pessaries and causing ulcerations, mayhap; and it may be proper to state here, (though I never said anything of the kind to the patient), that I think these things might produce abscess. I have cured some seven or eight cases of procidentia and prolapsus by the use of the abdominal supporter alone, during the past year. It is safe. It is comfortable for the patient, and is liable to do no injury, that I am aware of; it certainly cannot produce "pelvic abscess."

One fact in this case was as favorable as remarkable, that no fæcal matter found its way into the abscess through the fistula in ano, which existed. In most cases of this kind, I believe it would be advisable, (if the patient would consent), to open the abscess through the vagina, which might prevent its pointing in the rectum, where usually it is very difficult to heal. This might also have prevented the necessity, which afterwards arose, of opening it externally.

